# CADFFNet: a dual-branch neural network for non-destructive detection of cigar leaf moisture content during air-curing stage

**DOI:** 10.3389/fpls.2025.1698427

**Published:** 2025-11-05

**Authors:** Zhuoran Xing, Yaqi Shi, Yihao Pan, Kai Zhang, Zhenhua Wang, Bingyang Liu, Xiangdong Shi, Songshuang Ding

**Affiliations:** ^1^ College of Tobacco Science, National Tobacco Cultivation and Physiology and Biochemistry Research Center, Key Laboratory for Tobacco Cultivation of Tobacco Industry, Henan Agricultural University, Zhengzhou, China; ^2^ Anhui Fermented Food Engineering Research Center, School of Food and Biological Engineering, Hefei University of Technology, Hefei Anhui, China; ^3^ Tobacco Leaf Production Department, Zhangjiajie City Branch of Hunan Tobacco Company, Zhangjiajie Hunan, China

**Keywords:** convolution neural networks, cigar leaves, dual-view images, feature fusion, moisture content prediction

## Abstract

**Introduction:**

The cigar leaves moisture content (CLMC) is a critical parameter for controlling curing barn conditions. Along with the continuous advancement of deep learning (DL) technologies, convolutional neural networks (CNN) have provided a way of thinking for the non-destructive estimation of CLMC during the air-curing process. Nevertheless, relying merely on single-perspective imaging makes it difficult to comprehensively capture the complementary morphological features of the front and back sides of cigar leaves during the air-curing process.

**Methods:**

This study constructed a dual-view image dataset covering the air-curing process, and proposes a regression framework named CADFFNet (channel attention weight-based dual-branch feature fusion network) for the non-destructive estimation of CLMC during the curing process based on dual-view RGB images. Firstly, the model utilizes two independent and parallel ResNet as its backbone structure to capture the heterogeneous features of dual-view images. Secondly, the Dual Efficient Channel Attention (DECA) module is introduced to dynamically adjust the channel attention weights of the features, thereby facilitating interaction between the two branches. Lastly, a Multi-scale convolutional feature fusion (MSCFF) module is designed for the deep fusion of features from the front and back images to aggregate multi-scale features for robust regression.

**Results:**

On five-fold cross-validation, CADFFNet attains R2 of 0.974±0.007 and mean absolute error (MAE) of 3.80±0.37%. On an independent cross-region, cross-variety testing set, it maintains strong generalization (R2=0.899, MAE=5.82%), compared with the classic CNN models ResNet18, GoogLeNet, VGG19Net, DenseNet121, and MobileNetV2, its R2 value has increased by 0.047, 0.041, 0.055, 0.098, and 0.090 respectively.

**Discussion:**

Generally, the proposed CADFFNet offers an efficient and convenient method for non-destructive detection of CLMC, providing a theoretical basis for automating the air-curing process. It also provides a new perspective for moisture content prediction during the drying process of other crops, such as tea, asparagus, and mushrooms.

## 1 Introduction

As a significant economic crop, tobacco plays a vital role in enhancing farmers' income (Soneji et al., 2022). Notably, the consumption of hand-rolled cigars, a key tobacco product, has risen significantly in recent years (Yang et al., 2024a), driving an increasing demand for high-quality cigar leaf raw materials. The air-curing process is critical to the morphological changes and quality formation of cigar leaves (Zhao et al., 2024b). Among various factors, the CLMC is a vital parameter for controlling temperature and humidity in curing barns. Typically, operators estimate CLMC levels through visual inspection and tactile judgment, which highly rely on experience and are prone to inaccuracies. Conventional methods for quantifying moisture content, such as hot-air drying (Rahman et al., 2025) and Karl Fischer titration (Kosfeld et al., 2022), are complex, destructive, and incapable of providing real-time, non-invasive measurements. Recently, several novel indirect moisture content detection technologies have been proposed for agricultural products production, including spectral analysis (Guo et al., 2023; Liu et al., 2023), microwave technology (Jin et al., 2023), and nuclear magnetic resonance (NMR) (Qu et al., 2021). For instance, Wei et al. (2021) employed multispectral images of tea leaves to develop a moisture prediction model. Similarly, Yang et al. (2024b) collected spectral and morphological data of alfalfa seeds under varying moisture levels and distinguished them based on machine learning discriminant models. Moreover, Wu et al. (2022) utilized multifrequency microwave signals to detect tea leaves moisture content during the withering process. Despite these advancements enabling the non-invasive detection of agricultural products, methods such as moisture content extensively depend on expensive equipment, limiting their scalability in practical production scenarios. Therefore, the accurate, convenient, and non-destructive estimation of CLMC is crucial for improving the standardization level of the cigar leaf air-curing process.

With advancements in technology, computer vision has demonstrated significant potential in various aspects of tobacco production, including disease identification (Lin et al., 2022) and determining harvest maturity (Dai et al., 2024). Indeed, by training a model on extensive image datasets, it can learn the correlation between moisture content and the subtle changes in color, texture, and shape of leaves (Xing et al., 2025), enhancing the automation of tobacco leaf curing processes. For instance, Condorí et al. (2020) developed a high-precision prediction model to learn the relationship between color changes in tobacco leaf images and weight loss, enabling automated control of curing barns during the curing stage. Wu et al. (2014) utilized color features from tobacco leaf images to construct an artificial neural network model for predicting the temperature rise time during the bulk curing process. Furthermore, Pei et al. (2024) integrated data from stem weight sensors, temperature and humidity sensors, and digital cameras to introduce the comprehensive predictive curing model (CPBM), which facilitates stage recognition during the tobacco curing process. Traditional machine learning (ML) algorithms often require manual feature design, typically relying on domain-specific expertise and task-specific requirements. Nevertheless, such an approach often yields incomplete feature extraction, compromising model performance, particularly with complex and diverse image datasets (Shafik et al., 2024). In contrast, Deep Learning (DL) utilizes more complex neural networks to progressively transform input data into more abstract representations, enabling the model to automatically extract features without human intervention. Among them, convolutional neural networks (CNN) have shown exceptional performance in handling large-scale image data due to their inherent ability to learn features and their generalizability autonomously (Chen et al., 2023). In recent years, CNN-based solutions have been widely studied in the field of crop production (Xu et al., 2020; Wang et al., 2023). For example, Dai et al. (2025) designed a new feature extraction network structure, for determining lightness levels during various stages of tobacco curing, which facilitates the exchange of information between features at different image levels, thereby enhancing the model's classification accuracy. By integrating multi-scale features, the network effectively combines the global features and local features of the image, leading to improved performance. Zhao et al. (2024a) proposed a lightweight recognition model for tobacco leaf curing stages, named TCSRNet. By integrating Inception branches and Multi-scale Adaptive Attention Mechanism (MAAM), this model achieves a classification accuracy of approximately 90.35% with 158.136 MFLOPS and 1.749M parameters. Feng et al. (2025) compared the model's recognition accuracy under different image acquisition conditions during the tobacco curing process. The results indicated that combining images from multiple angles significantly improved the model's accuracy in determining the curing stage.

The above researches indicate that CNNs demonstrate applicability in the intensive curing process of flue-cured tobacco. However, the intensive curing process of flue-cured tobacco has a relatively short cycle, and each curing stage is equipped with its corresponding process parameters. In contrast, the air-curing process of cigar leaves is relatively slow, and the environmental temperature and humidity are not strictly controllable (Zhao et al., 2022). Therefore, the accurate estimation of moisture content during the air-curing process of cigar leaves has become a problem that urgently needs to be solved. For example, Yang et al. (2023) demonstrated that, compared to traditional machine learning algorithms, CNNs offer superior performance in predicting the CLMC during the air-curing process. Yin et al. (2024) used Visible and Near-infrared hyperspectral imaging (VNIR-HSI) combined with a Diversified Region-based Convolutional Neural Network (DR-CNN) to predict the CLMC during the curing process. The results showed that in terms of prediction accuracy, DR-CNN outperformed PLSR and traditional CNN models. Notably, current studies have primarily focused on the single-view image features of tobacco leaves (Gao et al., 2021; Wu et al., 2024). However, during the air-curing stage, as water lost, the leaves gradually curl inward, causing the front surface to be obscured by the back. Additionally, the vein of the leaf becomes fully exposed on the back surface. Consequently, relying solely on the single-view images for modeling entails a significant loss of crucial information.

To address the above issues, we propose a channel attention weight-based dual-branch feature fusion network (CADFFNet). Specifically, the contributions of this study are as follows:

- Construction of the cigar leaf air-curing process image dataset: To replicate the authentic curing process of cigar leaves, images of the cigar leaf in their naturally suspended state were used to predict moisture content.
- Design of the CADFFNet: The model employs two parallel ResNet-18 backbones to extract features from front- and back-view leaf images. A DECA module performs cross-view channel alignment, and an MSCFF module carries out multi-scale feature fusion. A subsequent regression head outputs the CLMC. By leveraging dual-view imagery, this design improves the predictive accuracy of CLMC estimation.
- Experimental Validation: The CADFFNet model successfully predicts the CLMC during the air-curing process, demonstrating robust performance across different planting regions and varieties.

The remainder of the paper is organized as follows. Section 2 describes the datasets constructed and the proposed method. Section 3 reports the experiment results, Section 4 discusses the advantages and limitations of the proposed methodology, and Section 5 concludes this work.

## 2 Materials and method

### 2.1 Datasets

**Figure 1:**
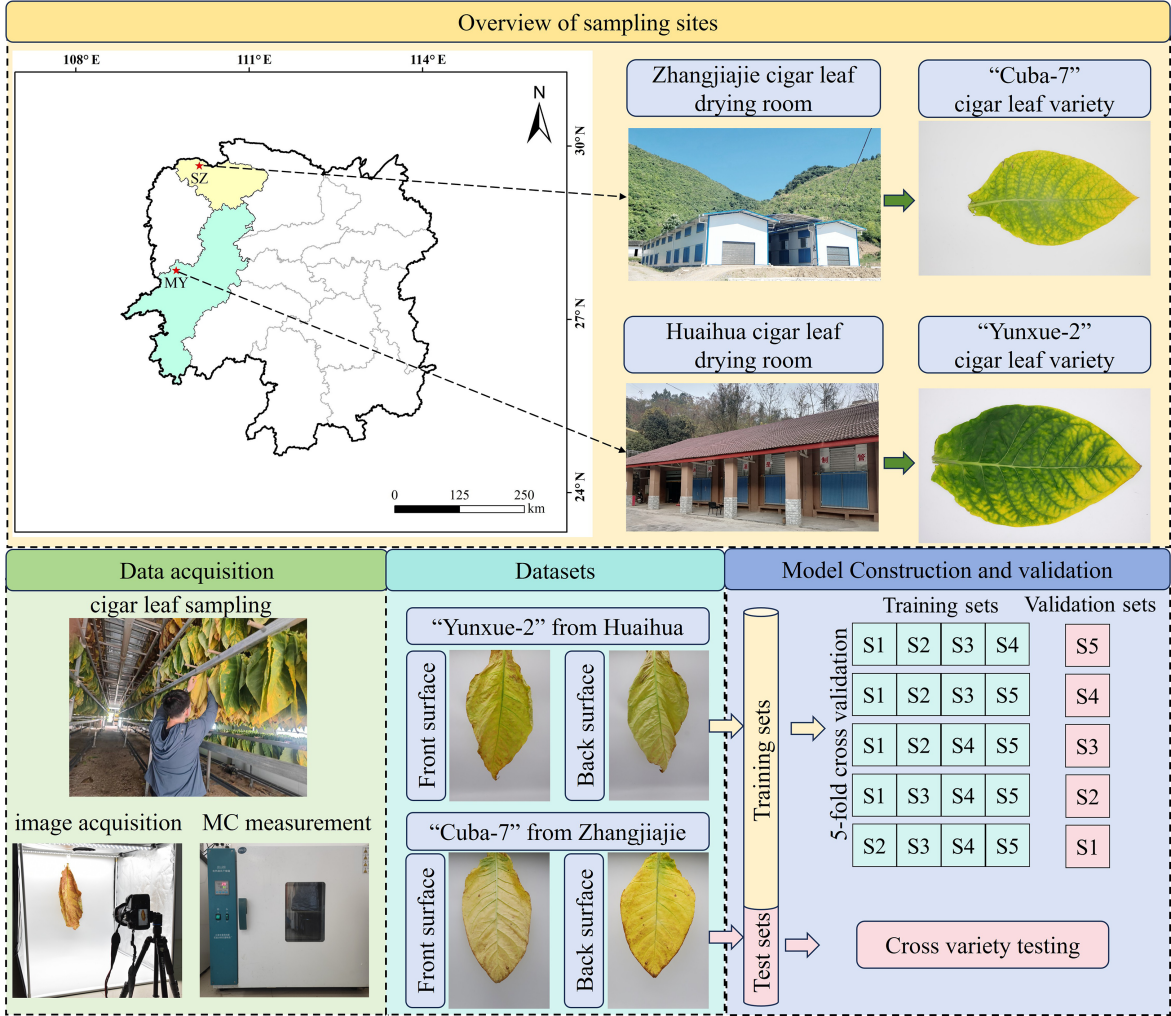
The roadmap of this study. (MY, MaYang; SZ, SangZhi).


**Figure 1** illustrates the data collection process of this study. After harvest, cigar leaves were fixed onto wooden rods using cotton threads for curing. To closely replicate the authentic curing process, the image collection procedure followed the method described in Ma et al. (2024). Specifically, after sampling the leaves, they were suspended in a dark chamber equipped for image capture. A digital camera (Canon Mark II, 24-70mm/f4 lens, focal length of 35 mm, 2,080×3,120 pixels resolution) was positioned 90 cm away from the leaves, capturing images of both the front and back surfaces. Inside the dark chamber, a white background board was placed, and two 40W lamps were positioned at the top to provide lighting. After the image was captured, the fresh weight of the leaves was immediately measured. Subsequently, the CLMC was determined using the hot-air drying method (Chen et al., 2021). The cigar leaves MC can be calculated as shown in Equation 1:

(1) $$MC(\%)=\frac{M_{fresh}-M_{dry}}{M_{fresh}}\times 100\%$$

where *MC* is the moisture content of cigar leaves, and *M_fresh_* and *M_dry_* are the fresh weight and dry weight of cigar leaves, respectively.

Where *MC* is the cigar leaves moisture content, and *M_fresh_* and *M_dry_* are the fresh weight and dry weight of cigar leaves, respectively.

The datasets used in this study were obtained from Mayang County, Huaihua City, Hunan Province (109°39′E, 27°54′N, at an altitude of 300 m). The data were gathered from the day of leaf harvest (0d) until the leaves were removed from the curing barn. The barn temperature and humidity parameters of the drying process are shown in **Table 1**. In total, 1,005 cigar leaves were collected, yielding 2,010 dual-view images for model training. To fully exploit the practical information contained in this small-scale dataset and enhance the reliability of model evaluation, we applied a 5-fold cross−validation scheme. Specifically, the entire dataset was randomly divided into 5 non−overlapping parts of roughly equal size, in each fold, one subset was used for validation, while the remaining four subsets were used for training, this rotation ensures that each sample served as the validation set exactly once, which enables a quantitative assessment of the model's robustness and stability across different data partitions (Niu et al., 2025).

**Table 1:** Key parameters of the cigar leaves air-curing process.

Air-curing period	Time (d)	Temperature (°C)	Humidity (%)
Wilting period	3-4	26-29	85-95
Yellowing period	4-7	27-32	80-90
Browning period	5-7	30-33	75-85
Fixation period	7-8	32-35	60-70
Dry tendon period	7-8	35-40	50-60

To further assess the model’s ability to generalize across diverse production regions and cigar varieties, an independent test set was additionally constructed. This test set comprised 175 front-side and 175 back-side images of the "Cuba-7" cigar leaves, collected from Sangzhi County, Zhangjiajie City, Hunan Province (110°16′E, 29°39′54″N; at an altitude of 308 m). The resolution of all images was resized to 224×224, and the corresponding CLMC labels were generated to match the model's input requirements and reduce computational complexity.

**Figure 2:**
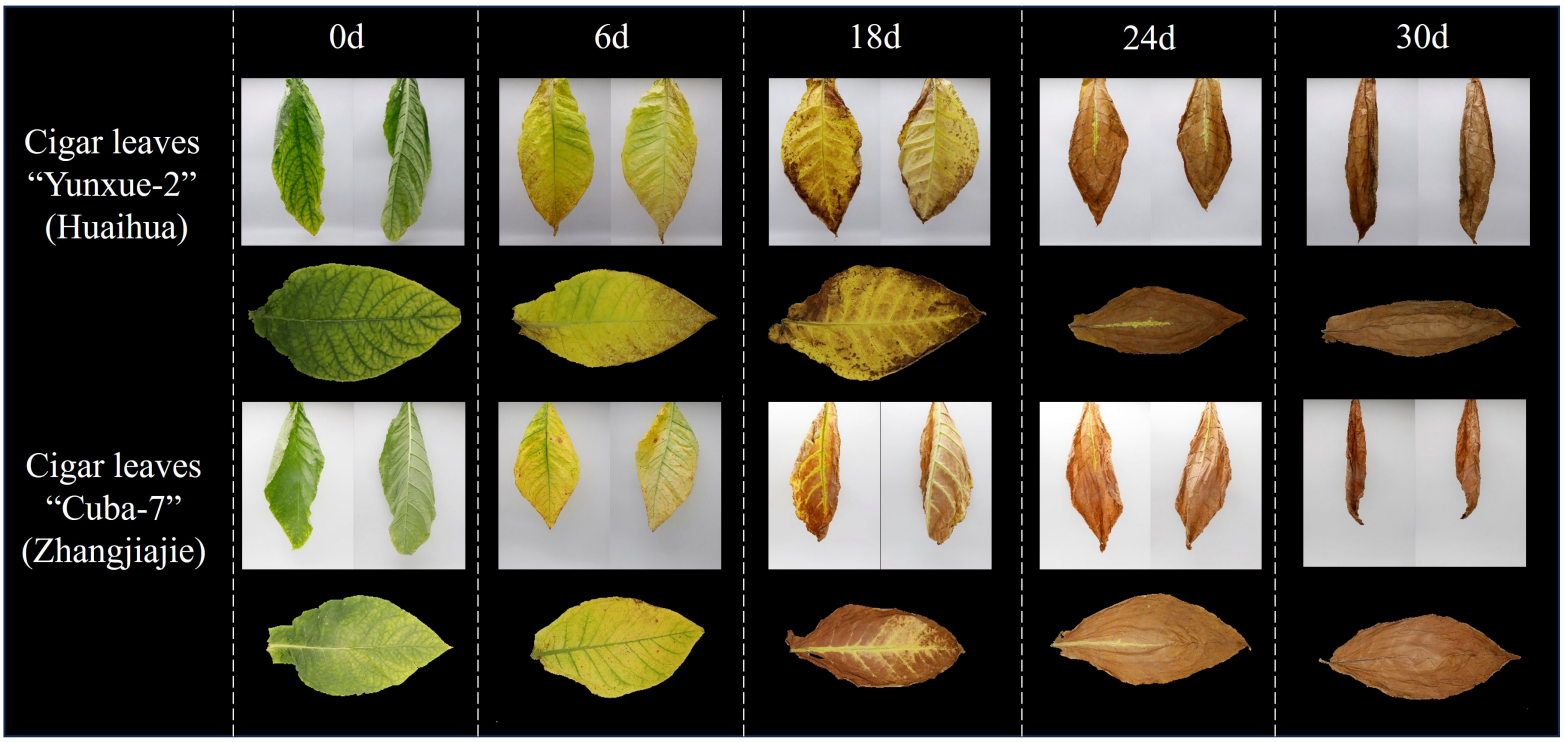
Phenotypic differences between different cigar varieties.

After harvest, the cigar leaves are initially green, as curing progresses, its color shifts to yellow and then deepens. As shown in **Figure 2**, the leaves undergo noticeable phenotypic changes during air-curing: surface color shifts, the lamina curls inward, and venation becomes increasingly exposed on the abaxial (back) surface. Consequently, dual-view (front–back) imaging captures more comprehensive information than single-view acquisition. In addition, Significant visual differences are observed in the leaves from different cultivation areas during curing, which are primarily attributed to the cigar variety and local climatic conditions (Jiang et al., 2024). Based on these differences, this study utilized images of cigar leaves from the Huaihua cultivation area for model training and validation. In contrast, photos from the Zhangjiajie cultivation area were employed to evaluate the model's performance, thereby testing its generalization ability.

### 2.2 Proposed CADFFNet

**Figure 3:**
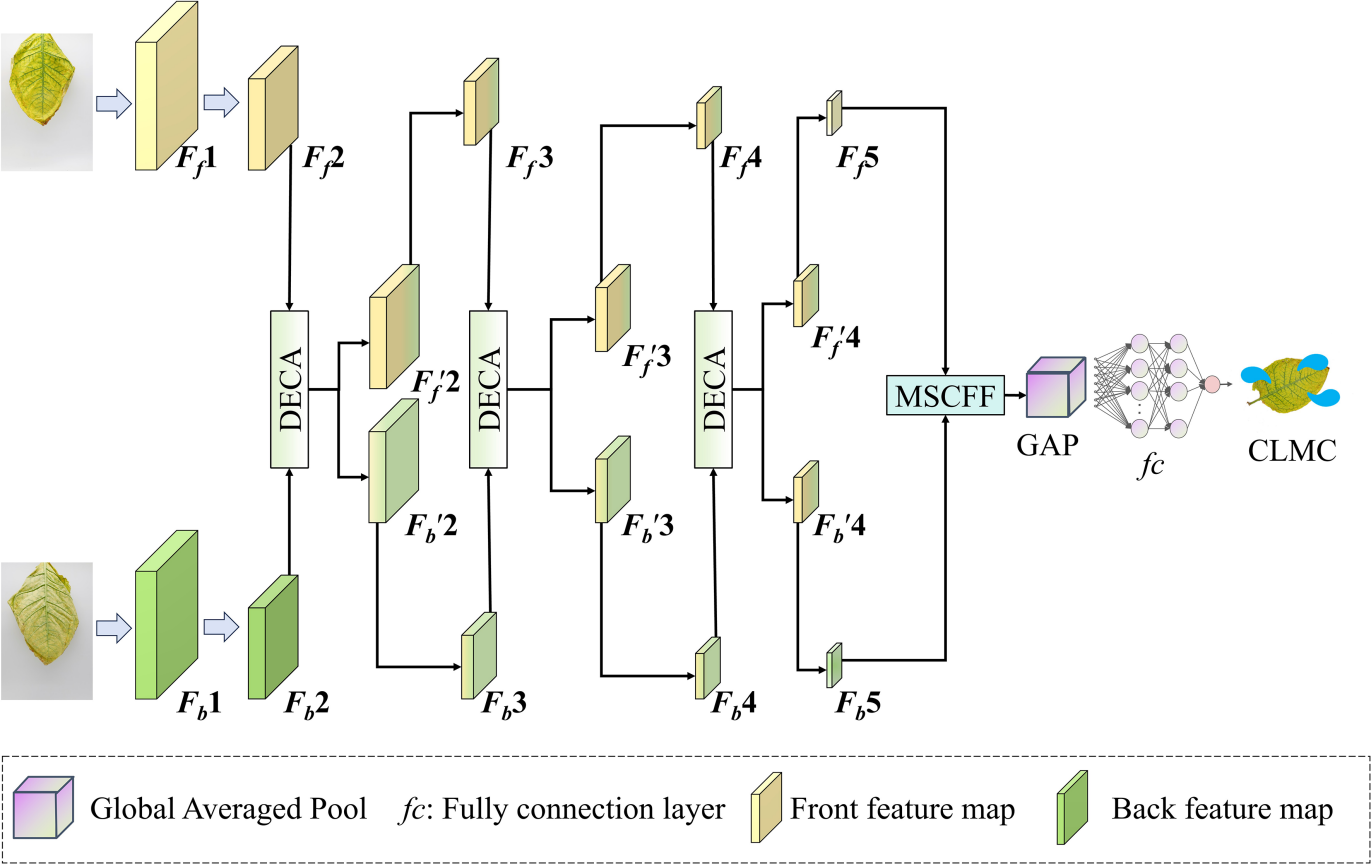
CADFFNet architecture.


During the CLMC prediction in the air-curing process, the dual-view images of the leaves often exhibit similar or complementary patterns. To obtain a comprehensive depiction of leaf morphology and exploit the complementary information from both surfaces, while the interaction between the two sides enhances the feature expression capability. This study introduces CADFFNet, as depicted in **Figure 3**, which comprises four modules: the front-side feature extraction branch, the back-side feature extraction branch, the DECA module, and the MSCFF module. The model relies on ResNet (He et al., 2016) as its backbone structure to extract image features. Specifically, the preprocessed front and back images are first input into the dual-branch feature extraction modules, each branch uses ResNet to derive hierarchical features from the front and back images independently. Then, the feature maps from three intermediate stages of the two branches are dynamically interacted with using the DECA module, which highlights key channel information and improves the network’s ability to capture common patterns on both sides. Subsequently, the feature maps output by Stage 5 of both extraction modules are concatenated along the channel dimension, and input to the MSCFF module to enhance multi−scale perception. Finally, followed by global pooling, and then input into an *fc* layer to predict the results. Since the CLMC prediction is a regression problem, the model is trained using mean squared error (MSE) as the loss function, which is shown in Equation 2:

(2) $$loss=\frac{1}{\text{n}}\sum_{i=1}^{\text{n}}{\left(\hat{{\text{y}}_{\text{i}}}{\text{-y}}_{\text{i}}\right)}^{2}$$

### 2.3 DECA module

Conventional convolution operations are inherently limited by the receptive field, preventing sufficient attention to key information in the image. To address this, the channel attention mechanism, such as the ECA module, has been widely adopted, where 1D convolutions are applied to model inter-channel dependencies with minimal computational overhead (Lee et al., 2024). By dynamically assigning weights to each channel, the model's expressive capability and predictive performance are enhanced.

**Figure 4:**
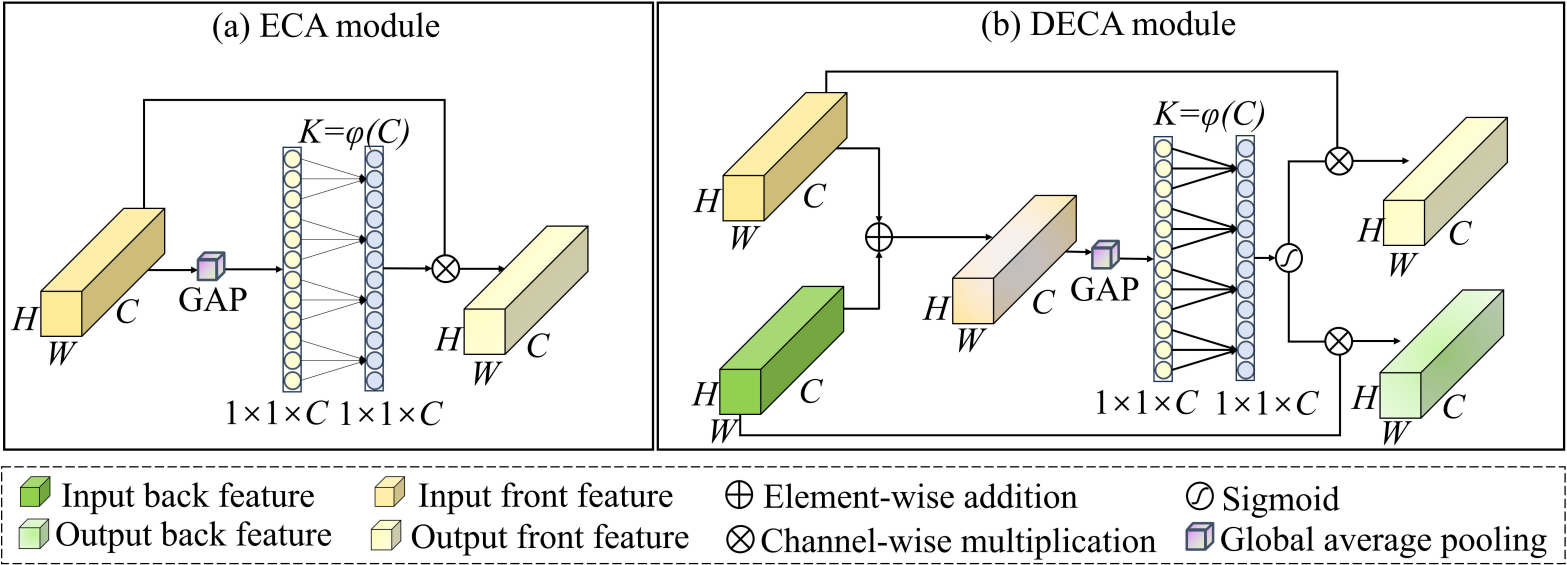
Structures of **(a)** ECA and **(b)** DECA modules.


Following the architecture of the ECA module depicted in **Figure 4a**, we devise a dual-branch variant, termed DECA, which is tailored to our two-stream network. DECA generates channel weight descriptors for the fused features and assigns them to the dual-branch network structure, enabling feature interaction between features and enhancing the model's response to similar pattern changes in both images (Wang et al., 2020). The corresponding architecture is depicted in **Figure 4b**. Given the feature map of front surface image *F_f_* ∈ R*^H × W × C^* and the back surface image *F_b_* ∈ R*^H × W × C^*, where *H*, *W*, and *C* denote the height, width, and number of channels of the feature map, respectively, the two maps are first fused by element-wise addition to obtain *P* ∈ R*^H × W × C^*. This process is shown in Equation 3:

(3) $$P=F_{f}+F_{b}$$

Subsequently, a global average pooling is applied to obtain the channel descriptor, which is then processed by a 1D convolution with a kernel size *K* to model local inter-channel interactions. The resulting vector is passed through a sigmoid activation to generate the channel-attention weights *W* which is shown in Equation 4:


(4) $$W=\;\sigma (\text{Conv}1D_{K}(AvePool(P)))$$

where the kernel size *K* is an adaptive parameter determined by the number of channels in the feature map. The calculation method of K is shown in Equation 5:


(5) $$K=|\frac{\log_{2}C+b}{\gamma }{|}_{odd}$$

where *γ* = 2, and *b* = 1. The term "odd" ensures that the kernel size is an odd number by rounding the absolute value to the nearest odd integer. Finally, the obtained channel attention weights are applied to the two input feature maps via channel-wise multiplication, resulting in the re-weighted feature maps $\hat{F_{f}}$ and $\hat{F_{b}}$ , which can be expressed as Equation 6:


(6) $$\left\{ \begin{array}{c} \begin{array}{c} \hat{F_{f}}=\;W⨀F_{f}\\ \hat{F_{b}}=\;W⨀F_{b} \end{array} \end{array}\right.$$

This module optimizes the channel weights of the fused front and back features, enhancing the model's ability on key patterns while suppressing the interference from redundant background information. Additionally, the module improves the independent feature representation of each branch and facilitates effective interaction between the front and back features, thereby further boosting the model's overall performance.

### 2.4 MSCFF module

**Figure 5:**
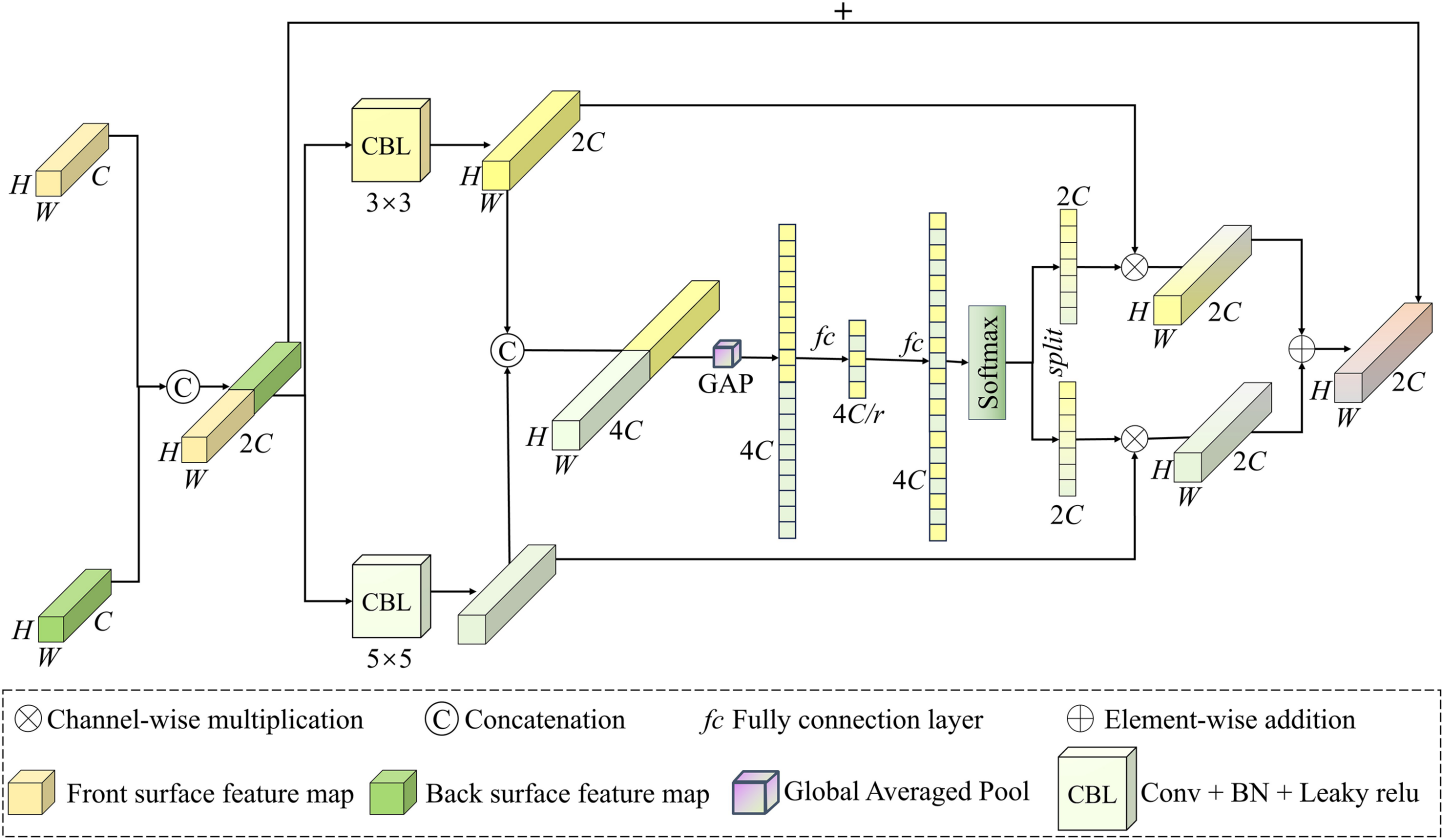
MSCFF module.


In order to effectively extract the different scale information of two feature maps, this study designs a multi-scale convolutional feature fusion module, inspired by the channel attention mechanism and multi-scale feature fusion concepts (Li et al., 2019). The MSCFF module architecture is illustrated in **Figure 5**, which comprises two convolutional kernels of different sizes, a global pooling layer, a fully connected layer, and a Softmax function. Let the front and the back surface feature maps be denoted as *F_f_* ∈ R*^H × W × C^* and *F_b_* ∈ R*^H × W × C^*, where *H*, *W*, and *C* denote the height, width, and number of channels of the feature map. First, the two feature maps are concatenated along the channel axis to obtain *Y* ∈ R*^H × W ×^* ^2^ *^C^*, which can be denote as Equation 7:

(7) $$Y=\;Concat\{F_{f};F_{b}\}$$

Then, two convolution kernels of different sizes are used to perform multi-scale feature extraction on *Y* to obtain feature representations under different receptive fields. The outputs of the two branches *Y*_1_, *Y*_2_ ∈ R*^H × W ×^* ^2^ *^C^* are then concatenated to obtain $\hat{Y}$ ∈ R*^H × W ×^* ^4^ *^C^* the calculation process is shown in Equations 8–10:


(8) $$Y_{1}=\;\sigma (BN(Conv_{3\times 3}(Y)))$$

(9) $$Y_{2}=\;\sigma (BN(Conv_{5\times 5}(Y)))$$

(10) $$\hat{Y}=\;Concat\{Y_{1};Y_{2}\}$$

Where *Conv*_3×3_ and *Conv*_5×5_ denote convolution kernels with sizes of 3 and 5, respectively, and the *BN* represents batch normalization, *σ* represents Leaky ReLU activation function. To coordinate the contributions of the two scale branches at the channel level, we apply global average pooling over the spatial dimensions of $\hat{Y}$ to obtain the channel descriptor *S* ∈ R^1^ *^×^* ^1^ *^×^* ^4^ *^C^*. The descriptor is then passed through two series fully connected layers with reduction ratio *r* (fixed to *r* =16 in this study) to obtain *S’* ∈ R^1^ *^×^* ^1^ *^×^* ^4^ *^C^*. Using ReLU function between the two layers, this process is shown in Equation 11:


(11) $$S^{\prime }=w_{2}*\;Relu(w_{1}*AvePool(\hat{Y}))$$

where $w_{1},w_{2}\in {\text{R}}^{\frac{4C}{r}\times 4C}$ , the channel reduction ratio is fixed to *r* =16 throughout this paper. a Softmax operation is applied to *S*′ along the channel dimension to produce a normalized weight vector, which is equally split into two parts *S*_1_, *S*_2_ ∈ R^1^ *^×^* ^1^ *^×^* ^2^ *^C^*. After broadcasting *S*_1_ and *S*_2_ to the spatial dimensions, element-wise multiplications with the corresponding scale features to obtain ${\hat{Y}}_{1},{\hat{Y}}_{2}$ ∈ R*^H × W ×^* ^2^ *^C^*. Finally, the two modulated feature maps are added element-wise and fused with the original *Y* through a residual connection to obtain the feature map *O* ∈ R*^H × W ×^* ^2^ *^C^*, which is shown in Equation 12:


(12) $$O=Y+S_{1}⨀{\hat{Y}}_{1}+S_{2}⨀\;{\hat{Y}}_{2}$$

## 3 Result

### 3.1 Experimental conditions and evaluation metrics

The experimental setup comprises a Core (TM) i7–12700 CPU, an NVIDIA GeForce RTX 3060 Ti with 16 GB of memory, configured with CUDA 12.6 for GPU-based training, and the operating system is Windows 10. The software environment is Python 3.9, utilizing the PyTorch 4.2.1 framework. The model training process uses the Adam optimizer, the batch size is set to 16, and the initial learning rate is 0.0001. The performance and generalization ability of CADFFNet for estimating CLMC during air curing are quantified using the coefficient of determination (*R* ^2^), root mean squared error (RMSE), and mean absolute error (MAE) as evaluation metrics, the calculation formulas are shown in Equations 13–15. *R* ^2^ reflects the overall goodness of fit of the model to the prediction results, RMSE indicates the degree of dispersion of prediction errors, and MAE represents the overall average error of the model's predictions. A higher *R* ^2^ value and lower RMSE and MAE values indicate that the model has better predictive performance.


(13) $$R^{2}=1-\frac{\sum_{i=1}^{n}{\left(\hat{y_{i}}-y_{i}\right)}^{2}}{\sum_{i=1}^{n}{\left(\bar{y}-y_{i}\right)}^{2}}$$

(14) $$\text{RMSE}(\%)=\sqrt{\frac{1}{n}\sum_{i=1}^{n}{\left(\hat{y_{i}}-y_{i}\right)}^{2}}$$

(15) $$\text{MAE}(\%)=\frac{1}{n}\sum_{i=1}^{n}|\hat{y_{i}}-y_{i}|$$

Where, *ŷ_i_* and *y_i_* represent the measured CLMC of the *i-*th and the model-predicted value, respectively, and *n* represents the total number of samples.

### 3.2 Prediction results of different model variants

Based on the number of network layers, four CADFFNet variants are constructed: CADFFNet18, CADFFNet34, CADFFNet50, and CADFFNet101. Each model is evaluated through 5-fold cross-validation and independently tested on an external dataset.

**Figure 6:**
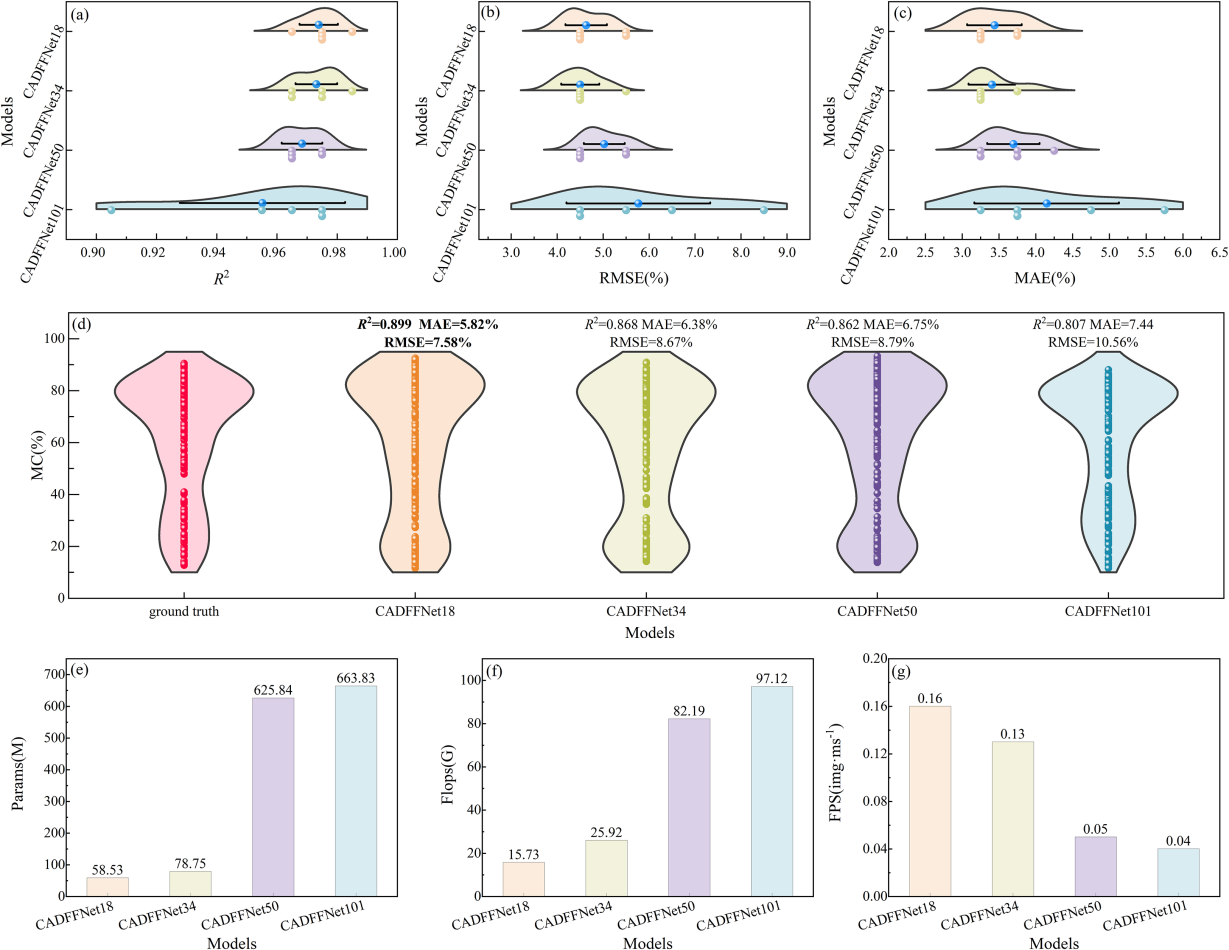
Performance comparison and complexity analysis of CADFFNet models with different layers under 5-fold cross-validation and independent testing. Subfigures **(a-c)** shows the distribution of *R* ^2^, RMSE, and MAE results from the five-fold cross-validation. The colored dots represent the evaluation metric values ​​for each fold for the four model variants. The box distribution is the kernel density estimate of the metric, and its width reflects the probability density of the result. Narrower boxes indicate less fluctuation between folds and greater stability. The blue dots and error bars represent the five-fold means and their 95% confidence intervals, respectively. The blue dots represent the five-fold cross-validation mean. Subfigure **(d)** shows the ground truth and the prediction results of different layers of models for the testing set, Subfigures **(e-g)** shows the parameter counts (Params), floating-point computation (Flops), and inference speed (FPS) of the four model variants, respectively.

**Figure 6** provides a comprehensive comparison of these four models, including their performance on cross-validation, prediction accuracy on the independent test set, and overall model complexity. **Figures 6a–c** highlight that CADFFNet18 outperforms all variants in terms of accuracy and consistency, in the five-fold cross-validation, the mean values of the *R* ^2^, MAE, and RMSE reached 0.974, 3.80%, and 4.63%, respectively, and there was less variation between each fold.

When increasing the network depth, CADFFNet34 and CADFFNet50 demonstrate a stronger representational capacity; however, their performance declines slightly due to overfitting on the limited dataset. In contrast, CADFFNet101 exhibits poor prediction performance and generalization ability, with mean values of *R* ^2^, MAE, and RMSE reached 0.955, 4.15%, and 5.77%, and standard deviations reached 0.027, 0.98%, and 1.56%, respectively. **Figure 6d** compares the distribution of predicted moisture content against the ground truth on the independent test set. Among all models, CADFFNet18 demonstrates the best generalization ability, with *R* ^2^, RMSE, and MAE values ​​reaching 0.899, 5.82%, and 7.58%, respectively. **Figures 6e–g** further illustrates that among the four model variants, CADFFNet18 achieves the best prediction performance and generalization ability while having fewer parameters, lower computational complexity, and faster inference speed.

In summary, CADFFNet18 offers the optimal balance between model accuracy and computational efficiency, outperforming deeper variants in both robustness and practicality. Therefore, we will only discuss CADFFNet18 in the following chapters of this research.

## 4 Discussion

### 4.1 Ablation experiment

An ablation study was conducted using the best-performing variant, CADFFNet18, on the constructed dataset to validate the effectiveness of the CADFFNet architecture. The single-branch ResNet18 models using front-view and back-view images were set as baseline models. Specifically, a dual-branch ResNet18 was used as the backbone, upon which the DECA and MSCFF modules were incrementally incorporated. Each configuration was evaluated on both the validation and independent test sets using five-fold cross-validation to assess predictive performance and generalization capability. **Table 2** summarizes the results, highlighting that the dual-branch variant significantly improves accuracy and robustness compared to the single-branch ResNet18 models. This demonstrates that jointly utilizing front and back-view images enhances the model’s ability to capture the structural characteristics of cigar leaves throughout the air−curing stage.

**Table 2:** Results of the ablation experiment.

Model	Cross-validation			Independent testing		
	*R* ^2^	MAE (%)	RMSE (%)	*R* ^2^	MAE (%)	RMSE (%)
Front image+ResNet18	0.868 ± 0.052	7.04 ± 1.87	7.06 ± 0.88	0.700	9.65	13.06
Back image+ResNet18	0.915 ± 0.028	5.81 ± 0.85	7.14 ± 0.97	0.728	9.64	12.44
Dual-branch ResNet18	0.968 ± 0.008	3.93 ± 0.43	4.88 ± 0.63	0.843	7.33	9.89
Dual-branch Resnet18+DECA	0.975 ± 0.007	3.44 ± 0.40	3.95 ± 0.57	0.856	7.09	9.01
Dual-branch ResNet18+MSCFF	0.968 ± 0.007	4.28 ± 0.37	4.87 ± 0.52	0.874	6.22	8.29
**CADFFNet**	**0.974 ± 0.006**	**3.80 ± 0.32**	**4.63 ± 0.45**	**0.899**	**5.82**	**7.58**

All evaluation metrics of CADFFNet constructed in this study are shown in bold font, the rest of the table remains the same.

Furthermore, introducing the DECA module further improves the predictive performance, suggesting its effectiveness in enhancing the network's ability to model symmetric and complementary patterns between dual-view features. Similarly, the MSCFF module contributes to better integration of the extracted multi-branch features. Notably, relative to the dual-branch ResNet-18 baseline, adding DECA alone outperforms adding MSCFF alone in five-fold cross-validation, whereas the independent test set shows the opposite trend. A plausible explanation is that DECA emphasizes channel-level alignment of complementary front and back features, thereby reducing bias under same source folds; in contrast, MSCFF focuses on multi-scale salient information and robustness, making it more sensitive to appearance and scale variations of cigar leaves during air-curing and thus suited on the independent test set.

Ultimately, integrating DECA and MSCFF into the full CADFFNet architecture achieves the best performance across all evaluation settings, with *R* ^2^, MAE, and RMSE reached 0.974 ± 0.007, 3.80 ± 0.37%, and 4.63 ± 0.45% in cross-validation, and 0.899, 5.82%, and 7.58% in the independent test set. These results confirm the complementary strengths of the two modules and highlight the overall effectiveness of the proposed network design.

### 4.2 Impact of the number of DECA modules on the model

The DECA module developed is placed at three key stages in the dual-branch network. To further explore the impact of the number and position of DECA modules on model performance, eight different configurations were constructed based on the number and insertion positions of DECA modules within the network. Their corresponding performance on five-fold cross-validation, the independent testing set is presented in **Table 3**.

**Table 3:** Impact of the number and location of DECA modules on the model.

Stage 2	Stage 3	Stage 4	Cross-validation			Independent testing		
			*R* ^2^	MAE (%)	RMSE (%)	*R* ^2^	MAE (%)	RMSE (%)
–	–	–	0.968 ± 0.007	4.28 ± 0.37	4.87 ± 0.52	0.874	6.22	8.29
✓	–	–	0.970 ± 0.008	3.89 ± 0.64	4.83 ± 0.42	0.877	6.25	8.36
–	✓	–	0.971 ± 0.007	3.83 ± 0.93	4.86 ± 0.57	0.878	6.04	7.69
–	–	✓	0.970 ± 0.007	3.92 ± 0.45	5.48 ± 0.64	0.878	6.13	8.27
✓	✓	–	0.971 ± 0.007	3.80 ± 0.39	4.69 ± 0.48	0.880	6.12	8.23
–	✓	✓	0.972 ± 0.006	3.87 ± 0.41	5.22 ± 0.39	0.883	6.05	7.75
✓	–	✓	0.972 ± 0.006	3.81 ± 0.44	4.69 ± 0.51	0.884	5.96	7.74
✓	✓	✓	**0.974 ± 0.006**	**3.80 ± 0.37**	**4.63 ± 0.45**	**0.899**	**5.82**	**7.58**

All evaluation metrics of CADFFNet constructed in this study are shown in bold font, the rest of the table remains the same.

During cross-validation, all configurations exhibited similar predictive performance, with *R* ^2^ values ranging from 0.968 to 0.974 and MAE values ranging from 3.80% to 4.28%, indicating stable model behavior under limited data conditions. However, noticeable differences emerged in the independent testing results.

Specifically, when a single DECA module was inserted, the model's prediction accuracy on the test set improved compared to the baseline, and the module's insertion position had a minimal effect on performance. Further improvements were observed when two DECA modules were incorporated, with test set *R* ^2^ values reaching 0.880, 0.883, and 0.884, depending on the placement strategy. The best performance was achieved when all three DECA modules were inserted into the network.

These results suggest that inserting multiple DECA modules at different levels of the dual-branch architecture facilitates the interaction and fusion of multi-scale features. Hence, this design enhances the model's ability to capture low-level features, such as edges and textures, from both front and back views, and improves its understanding of high-level, abstract representations. Consequently, the model's predictive performance and generalization capability are further enhanced. The potential reason for this is that when multiple channel attention modules are inserted at different positions in the dual-branch model, they facilitate the interaction and fusion of feature maps at various scales. This enables the model to capture correlations between shallow features, such as edges and textures, of the front and back images, thereby improving its understanding of the deeper, abstract information in these images and enhancing its predictive performance and generalization ability.

### 4.3 Impact of different feature fusion strategies on the model

To verify the advantages of the MSCFF module in the feature fusion stage, we replaced it with three popular attention modules for comparison: Squeeze-and-Excitation (SE), Convolutional Block Attention Module (CBAM), and Coordinate Attention (CA). **Table 4** highlights that the proposed MSCFF module demonstrates the highest predictive performance and the lowest standard deviation during the five-fold cross-validation, and also achieves the best accuracy on the independent test set. The CA module attains the second-best performance, while the SE module yields the poorest results.

**Table 4:** Experimental results of different attention mechanisms.

Attention module	Cross-validation			Independent testing		
	*R* ^2^	MAE (%)	RMSE (%)	*R* ^2^	MAE (%)	RMSE (%)
SE (Hu et al., 2018)	0.974 ± 0.007	3.52 ± 0.48	4.78 ± 0.54	0.868	7.12	8.71
CBAM (Woo et al., 2018)	0.969 ± 0.006	4.04 ± 0.34	5.10 ± 0.44	0.869	6.90	8.54
CA (Hou et al., 2021)	0.974 ± 0.007	3.54 ± 0.45	4.89 ± 0.54	0.872	6.18	8.37
MSCFF (ours)	**0.974 ± 0.006**	**3.80 ± 0.37**	**4.63 ± 0.45**	**0.899**	**5.82**	**7.58**

All evaluation metrics of CADFFNet constructed in this study are shown in bold font, the rest of the table remains the same.

These findings suggest that constructing multi-scale features in combination with adaptive channel weight assignment significantly improves the network’s capacity to integrate both local and global information from feature maps. Thus, this combination effectively improves the model’s predictive performance and generalization ability.

### 4.4 Comparison with other CNN models

We compared CADFFNet against dual-branch network models with different neural network architectures as backbones for predicting cigar leaf moisture content during the curing process. The models employed were ResNet18 (He et al., 2016), GoogLeNet (Szegedy et al., 2016), VGG19Net (Simonyan and Zisserman, 2015), DenseNet121 (Huang et al., 2017), and MobileNetV2 (Sandler et al., 2018).

**Figure 7:**
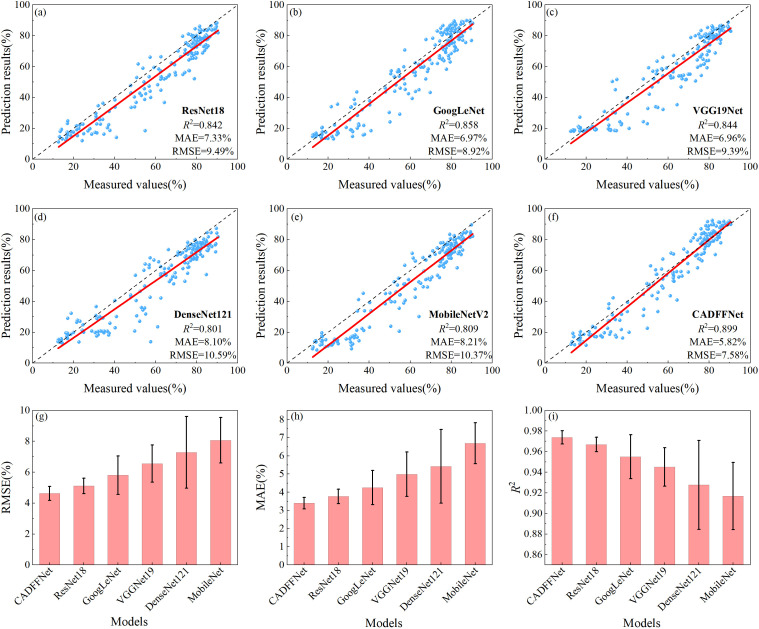
Performance comparison of different CNN models. Subfigures **(a-f)** prediction results of various models on the independent testing, Subfigures **(g-i)** comparison of *R* ^2^and MAE values obtained from five-fold cross-validation for each model.

**Figures 7a–f** present the prediction results of different models on the independent test set. The results demonstrate that CADFFNet achieves the highest agreement between predicted and measured moisture content values of cigar leaves, with the highest *R* ^2^ and the lowest MAE and RMSE, significantly outperforming other CNN-based architectures. Among the remaining five models, GoogLeNet exhibits the best predictive performance, followed by VGG19Net, which indirectly validates the effectiveness of multi-scale feature fusion and residual connections. In contrast, DenseNet121 provides the weakest results, with *R* ^2^, MAE, and RMSE of 0.801, 8.10%, and 10.59%.

**Figures 7g–i** showcase the *R* ^2^, MAE, and RMSE values of each model under five-fold cross-validation. The results highlight that CADFFNet exhibits the highest robustness and generalization capability, further confirming the superiority of the proposed architecture.

### 4.5 Comparison with other researches

In this study, we develop a dual-branch CNN model that dynamically perceives and fuses the features of dual-view images of cigar leaves to enhance the model's performance. To evaluate the performance of CADFFNet, we compared our results with those of current methods in this domain, using different original data and methods. Specifically, we evaluated the prediction performance of CADFFNet in comparison to Yin et al. (2024) and Yang et al. (2023), as reported in **Table 5**. The results demonstrate that CADFFNet outperforms both approaches in terms of CLMC prediction accuracy. This improvement suggests that the dual-branch network model can better capture the changes in the apparent morphology of cigar leaves resulting from water loss during the air-curing stage. Overall, our findings demonstrate that CADFFNet can efficiently and accurately predict CLMC, achieving high accuracy and better generalization ability.

**Table 5:** Algorithm comparison.

Related studies	Image type	Model	Source of test set	*R* ^2^
Yin et al. (2024)	Hyperspectral image of cigar leaves	Diversified Region-based CNN (DR-CNN)	Divided from the original data set	0.810
Yang et al. (2023)	RGB image of cigar leaves	CNN	Different curing barn	0.867
Ours	Front and back image of cigar leaves in suspend state	CADFFNet	Different planting region	0.899

### 4.6 Limitation and research prospect

During the air-curing process, cigar leaves change color and morphology due to water loss, with the operators assessing the leaf's appearance to estimate leaf moisture content (Fu et al., 2024). Accurately and non-destructively estimating the CLMC during the air-curing stage allows technicians to monitor the leaf's condition better and make more precise decisions regarding timely adjustments to the air-curing technique. Despite the strong predictive performance achieved by the proposed model, certain limitations remain. Firstly, as illustrated in **Figure 6**, when applied to cigar tobacco leaf samples from different production regions and varieties, the model still faces challenges in accurately predicting CLMC, suggesting that its generalization ability under heterogeneous data conditions requires further improvement. Secondly, due to the dual-branch architecture that necessitates parallel processing of two input images, the model—while benefiting from enhanced accuracy and robustness—inevitably incurs increased computational overhead. Finally, this study is based on static imaging of individual leaves, whereas in actual production cigar leaves are hung on whole stalks and air-cured at room scale. *In-situ* image acquisition in such settings faces occlusions and illumination variations, and therefore a gap remains to practical deployment.

The future research is multi-folded. First, we will collect cigar leaf images from multiple regions and varieties to expand the dataset, and utilize annual curing data for model iteration and updates to further enhance its robustness. Second, we will incorporate prior knowledge, such as fresh cigar leaf quality, barn temperature, humidity, and curing duration, into the model training process, and conduct continuous, high-frequency, multi-view *in-situ* imaging of whole-stalk leaves throughout curing. Based on these data, we aim to develop a non-destructive predictive model of the moisture-content–time trajectory over the curing process. Finally, another priority is to explore the correlation between cigar leaf images and chemical indicators, such as total sugars and nicotine content, as well as other chemical characteristics that directly characterize the quality of the cigar leaf, to further optimize the cigar leaf air-curing process.

## 5 Conclusion

This study introduces CADFFNet, a novel CNN model for non-destructive CLMC detection during the curing process, which integrates dual-view images of the cigar leaves. To better capture the similar pattern changes in the front and back images, we employ a dual-branch network structure to process the images in parallel. Additionally, we design the DECA and MSCFF modules, enabling feature maps at different levels to interact and fuse at various stages of the network. Moreover, introducing a channel attention mechanism allows the model to enhance key features of the leaves while suppressing irrelevant information that may interfere with the prediction results. Experimental results show that CADFFNet achieves excellent prediction performance on cigar leaves from the same region and variety. A strong performance is also demonstrated in cross-region and cross-variety predictions, with *R* ^2^ and MAE values of 0.899 and 5.82%, respectively, on the test set.

In summary, this study provides a convenient and non-destructive method for detecting CLMC, offering a theoretical basis for the automation of the cigar leaf air-curing process. Furthermore, the proposed approach, which integrates the interaction and fusion of front and back leaf images, provides a novel solution for pattern recognition tasks in plant leaves, such as leaf disease identification, crop classification, and assessment of plant growth stages.

## Data Availability

The raw data supporting the conclusions of this article will be made available by the authors, without undue reservation.
